# The Impact of Diet and Exercise on Drug Responses

**DOI:** 10.3390/ijms22147692

**Published:** 2021-07-19

**Authors:** Ellen Niederberger, Michael J. Parnham

**Affiliations:** 1 Pharmazentrum Frankfurt/ZAFES, Faculty of Medicine, Institute of Clinical Pharmacology, Goethe University Frankfurt, Theodor Stern Kai 7, 60590 Frankfurt, Germany; 2 Pharmacology Consultant, 65812 Bad Soden, Germany; mike.j.parnham@gmail.com; 3 EpiEndoPharmaceuticals ehf, Eidistorg 13, 170 Seltjanarnes, Iceland

**Keywords:** drug, pharmacokinetics, pharmacodynamics, exercise, diet

## Abstract

It is well known that lifestyle changes can alter several physiological functions in the human body. For exercise and diet, these effects are used sensibly in basic therapies, as in cardiovascular diseases. However, the physiological changes induced by exercise and a modified diet also have the capacity to influence the efficacy and toxicity of several drugs, mainly by affecting different pharmacokinetic mechanisms. This pharmacological plasticity is not clinically relevant in all cases but might play an important role in altering the effects of very common drugs, particularly drugs with a narrow therapeutic window. Therefore, with this review, we provide insights into possible food–drug and exercise–drug interactions to sharpen awareness of the potential occurrence of such effects.

## 1. Introduction

Pharmacological therapy of several different diseases is an important measure for health care systems to reduce mortality and morbidity and to increase the quality of life for patients. A key prerequisite for successful therapy is the efficacy and safety of the drugs used. These parameters are tested in large clinical trials before new drugs are approved. Although this is an accepted process and works well, it has to be taken into account that several factors may alter the drugs’ characteristics when used in the general population and lead to pharmacological plasticity [1]. Mutations in the drug target, leading to changes in protein expression or resistance mechanisms are one of these factors. This is the case, for instance, with G-protein coupled receptors (GPCRs) which are widespread drug targets and frequently show mutations [2]. Mutations may be induced by the drugs themselves, as in chemotherapy or, in other cases, by causing the organism to modify the drug [3]. The result, in both cases, is modulation of the patient’s drug response. Lifestyle changes may also alter the efficacy of drugs. Aging, smoking, physical activity as well as the composition of an individual’s diet and body mass index induce mechanisms which modulate the pharmacokinetics and pharmacodynamics of drugs [4,5,6,7,8,9].

A healthy diet and exercise are often suggested as beneficial approaches to avoid or cure several diseases. This assumption has been proven in the therapy of type 2 diabetes mellitus, dyslipidemia or cardiovascular disturbances [10,11] (https://www.who.int/health-topics/healthy-diet, accessed on 19 May 2021). However, in many cases, in particular in elderly patients or patients with chronic diseases, a basic therapy with adjusted diet and physical activity is not sufficient to achieve a satisfying therapeutic outcome. Therefore, patients are often placed under additional drug therapy. Since, as mentioned above, exercise and diet are able to modify drug responses, the combination of basic and pharmacological therapy might cause mutual interference, leading to exercise–drug or diet–drug interactions which are similar to drug–drug interactions. Several studies have shown that diet and body composition as well as exercise are lifestyle components that can influence many components of drug metabolism and efficacy [5,12,13]. Patients are often not aware of the potential risk factors, which are particularly important if drugs with a narrow therapeutic range are used. In this review, we will summarize effects of diet, nutritional state and exercise on the pharmacological plasticity of drugs and will discuss their clinical significance.

## 2. Drug Pharmacokinetics and Metabolism

Therapeutic drugs are subject to mechanisms of pharmacokinetics (PK) and pharmacodynamics (PD) which determine the disposition of the drug in the body (related to drug absorption, distribution, metabolism and elimination (ADME)) and its pharmacological effects (related to its mechanism of action), respectively. It is obvious that changes in these PK/PD parameters can influence the efficacy of the drug as well as its side effects and toxicity.

**Figure 1:**
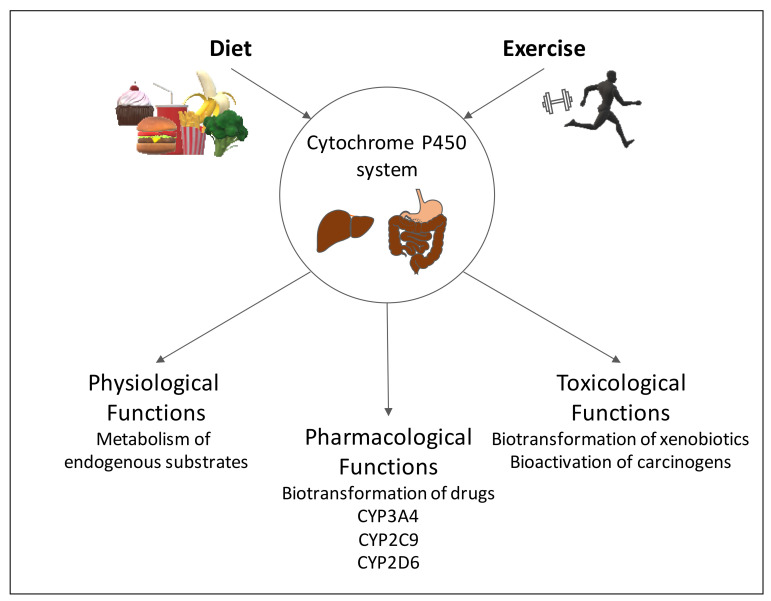
Schematic overview of the influence of diet and exercise on the human CYP450 system.

Absorption is dependent on the administration route of the respective drugs. The most common route of drug administration is orally since it is most convenient for physicians and patients. Most orally-used drugs are absorbed in the gastrointestinal tract (GI). The intestine already contributes to metabolism and elimination of some drugs, while several medications are transferred to the liver in an unchanged manner. Some drugs are then directly transferred to the systemic circulation to reach their target tissue while others are metabolized in a 2-step reaction. Phase I is the biotransformation reaction by hydroxylation, nitrosylation or sulfonation which is followed by phase II, a conjugation step leading for example to glucuronidation of the drug. The most important metabolic enzyme family in phase I is the cytochrome P450 system (CYP) (Figure 1) expressed in liver, gastrointestinal tract and other tissues. These enzymes comprise a variety of isoenzymes and are able to either bioactivate or metabolize drugs in the so-called first pass metabolism (mainly CYP3A4 (~45–60% of all drugs) and CYP2D6 (~30% of all drugs)) [14,15,16]. CYP450 activity is particularly interesting for the determination of drug interactions since several drugs are CYP inducers or inhibitors and also food ingredients are able to alter CYP activity and thereby, the drug’s pharmacodynamics. Further important players in drug absorption and elimination are transport proteins such as P-glycoproteins (Pgp) or organic anion transporter peptides (OATPs) which are mainly located in the intestines. Pgps are efflux transporters contributing to a lowering of drug concentrations while OATPs are transmembrane proteins which facilitate the uptake of drugs.

The rate and extent of absorption, metabolism and elimination are important parameters for the bioavailability (F) of a drug which indicates the amount of drug that reaches its target site of action. Bioavailability can be determined by calculating the area under the curve (AUC) of peak plasma concentrations (C_max_) and the time to reach peak plasma concentration (T_max_). For intravenously (i.v.) administered drugs, F is 100% (F = 1). The determination of the AUC of orally administered drugs in relation to the AUC of i.v. drugs allows the calculation of their absolute oral bioavailability (F_oral_). In addition, the elimination (drug clearance, CL), characterized by the half-life (t_1/2_) of a drug in plasma, has an impact on the bioavailability. The rate and extent of drug absorption can be modulated by many factors, such as expression and activity of drug transporter proteins or first-pass metabolism, respectively. The liver is one of the most important metabolic organs. Therefore, changes in liver function caused by altered hepatic blood flow or metabolic capacity can lead to a variability of drug effects. The dietary composition, meaning the quantity of proteins, carbohydrates and fat, but also vitamins and minerals in a meal, contributes to metabolic function, thus indicating that fasting and overnutrition can influence metabolism. In addition, exercise, which leads to alteration in liver blood flow, may have an impact on drug metabolism.

## 3. Diet

A balanced diet is recommended as a basal non-pharmacological measure for the treatment of several diseases, including diabetes or disturbances in lipid metabolism (https://www.who.int/health-topics/healthy-diet, accessed on 19 May 2021). Furthermore, several components of food, drinks and supplemental vitamins and minerals are suggested to support a healthy life. Many dietary products influence a wide variety of different cell types and their physiological signal transduction mechanisms and can thereby, affect their function. In addition, there is increasing knowledge on the pharmacological impact of different food ingredients. Food intake as well as the nutritional status of patients can drastically alter the therapeutic efficacy of drugs which can be of considerable relevance for therapy outcome and the potential for unwanted side effects. Bioavailability, as one of the most important variables for drug efficacy and toxicity is affected by drug absorption and drug metabolism. The amount, composition and also the time point of a meal potentially alters pharmacokinetic processes, either by direct drug–food interaction, by physiological reactions including changes in GI pH, GI motility or bile acid secretion and also by biochemical mechanisms (Table 1). Moreover, the individual body mass (body composition) influences drug pharmacokinetics with respect to absorption, distribution and metabolism of the medication [17,18,19].

### 3.1. The Impact of Body Weight

Body weight and obesity are known to be important factors in drug metabolism. Increase in body weight as a result of increased muscle mass is generally a healthy approach as the percentage of fat decreases. Obese people have a higher mass of fat and of lean tissue; however, the percentage of lean tissue per kg total body weight is reduced compared to normal-weight persons [26]. In addition to an increase in adipose tissue, the massive increase in body weight alters physiological functions such as cardiac output, as well as changes in blood flow distribution and body water which contribute to modified pharmacokinetics of drugs in obese individuals [18]. It has already been suggested that drug therapy has to be adjusted in these people, but specific recommendations are generally lacking and therefore, careful therapeutic drug monitoring is necessary in obese patients [27]. Absorption after oral administration shows no significant changes in these patients [18], but due to an increase in subcutaneous adipose tissue, drug absorption after subcutaneous or intramuscular administration might be changed [28,29]. Drug distribution and bioavailability cannot be directly predicted by body changes in obese individuals but strongly depend on the physicochemical properties of the respective drugs [18]. In the last 50 years or so, there have been a lot of clinical trials on CYP-mediated drug metabolism in obese and normal weight individuals, as well as effects of dietary compositions on the pharmacokinetics of different drug classes [7].

Cytochrome P450 enzymes constitute the best-known pharmacokinetic system which is responsible for the metabolism of about 70–80% of the drugs on the market. The liver is the major site of CYP450 expression and activity, but enterocytes in the epithelium of the small intestine are also involved in CYP-mediated metabolism. The major CYP enzymes contributing to drug metabolism comprise CYP1A2, CYP2C9, CYP2C19, CYP2D6 and CYP3A4 with CYP3A4 accounting for about 45–60% of drug metabolism, for instance that of statins, benzodiazepines or chemotherapeutics [14,15,16]. The activity of these enzymes is considerably affected by body weight and obesity with CYP3A4 activity being reduced and CYP2E1 activity increased by obesity, as indicated, for instance, by reduced clearance of alfentanyl or triazolam (Cyp3A4 substrates) and an increase in clearance of halothane or chlorzoxazone (Cyp2E1 substrates). So far, data on CYP1A2, CYP2D6 and CYP2C19 activities are inconclusive, although a tendency towards increased activity has been observed [30,31].

To date, less is known about effects of severe underweight on the CYP system. A clinical study investigating the activity of CYP1A2, CYP2C9, CYP2C19, CYP2D6 and CYP3A4 in patients with anorexia nervosa, again delivered hints that the body mass index (BMI) correlates with the metabolic activities of the enzymes. The metabolic activity of CYP3A4 decreased, while CYP1A2 increased at a higher BMI. CYP2C9, CYP2C19 and CYP2D6 remained unaltered [32], indicating that CYP3A4 substrates, such as statins or Cyp1A2 substrates like theophylline, might show differences in their efficacy or toxicity, respectively. A further study supported these data showing higher CYP3A4 activity in underweight patients in comparison to normal-weight controls [33]. These results indicate an inverse relationship between body weight and CYP3A4 activity which might have clinical relevance for specific drug therapies. The determination of renal clearance in obese patients is again difficult and depends on the extent of kidney function which is often disturbed in these people [34].

### 3.2. The Impact of Nutrition

The content and composition of meals with regard to macro- and micronutrients, as well as timing of meals in relation to drug uptake, have a strong influence on the metabolism and bioavailability of drugs [35,36]. These food–drug interactions have already been known for a long time and are as important as drug–drug interactions. Some medications are affected by food uptake in general, with a simultaneous meal leading to an increased or decreased absorption of the drug, irrespective of the meal composition and largely dependent on the physiochemical properties of the drugs (see Table 1). Therefore, co-administration of drugs and food may be associated with increased bioavailability of a drug, thereby enhancing its efficacy but also its potential side effects. On the other hand, food can decrease the absorption of drugs, reduce their bioavailability and thereby the efficacy of the therapy. Other drugs react with certain food components like proteins (for example, propranolol), fat (for example, cyclosporine), carbohydrates (for example, theophylline) or vitamins (for example, warfarin) or specific types of meals [37]. These potential interactions already show that it is important to raise the awareness of patients and doctors and to consider potential food–drug interactions in the development of new medicines in the future.

Taking this problem into account, the FDA recently published recommendations for the development of novel therapeutics (2019, https://www.fda.gov/media/121313/download, accessed on 19 May 2021). According to these guidelines, pharmacokinetic parameters of new drugs including AUC, C_max_, T_max_ and T_1/2_, clearance should be determined in clinical studies in individuals under different nutrition states. It is suggested that a high fat-high calorie meal will have the biggest effect on drug pharmacokinetic properties. Nevertheless, these recommendations do not by any means cover the whole spectrum of dietary habits of patients. Furthermore, it makes a difference if meals are taken only occasionally or if they represent prolonged dietary changes. A huge number of studies has been performed so far to assess effects of diet on a variety of different drugs. Therefore, in this review, it is only possible to give some examples of food–drug or nutrient–drug interactions and not to cover the whole range of potential interactions.

#### 3.2.1. Effects of Macronutrients

Macronutrients are carbohydrates, lipids and proteins in food serving primarily as an energy supply. In addition, these food ingredients have different impacts on drug metabolism. Fat-, protein- and carbohydrate-content of a meal differentially alters the properties of drugs and their metabolism.

In general, high-fat food leads to lowered gastric emptying [38]. The solubility of hydrophilic drugs can be decreased while a high fat content can increase the solubility of hydrophobic drugs which, for instance, is the case for antifungal itraconazole (lipophilic) or the beta-blocker atenolol (hydrophilic), showing increased and decreased bioavailability after high fat meals, respectively [39,40]. Furthermore, high-fat food alters several physiological processes such as bile acid secretion, intestinal lymphatic transport pathways and transporters in the gut [41]. Thus, high-fat diets have been shown to alter the disposition of cyclosporine by increasing its clearance [42]. Such diets also have selective effects on drug metabolizing enzymes. In a recent study on five different drugs, each metabolized by a different CYP, it was shown that the high fat diet increased exposure to the benzodiazepine sedative midazolam and gastric proton pump inhibitor omeprazole, indicating modulation of CYP3A4 and CYP2C19, respectively [43]. This is further confirmation of the fact, discussed above, that these two CYPs are targeted by lifestyle changes in metabolism, though the effects of high fat intake appear to differ from those of obesity.

Food contains different types of fat which can be roughly divided into unsaturated (mono- or polyunsaturated) and saturated fats. Several essential polyunsaturated fatty acids (PUFAs), such as linoleic or arachidonic acid, are metabolized by cytochrome P450 epoxygenases to biologically active epoxyeicosatrienoic acids (EETs) [44]. PUFAs are associated with a number of beneficial health effects, particularly due to their anti-inflammatory properties, but there are also hints that they may interact with certain drugs [45]. However, despite clear evidence of interactions with CYPs and an immense literature on the effects of dietary PUFAs on health and disease, there appear to be no or very few studies on the effects of a specific PUFA diet on drug metabolism. On the other hand, it has been shown that pharmacodynamic properties can be altered by specific types of fats.

PUFAs constitute components of cell membranes and it has long been known that an increase in PUFA intake results in enhanced fluidity of biological membranes [46]. In view of the fact that receptors for drugs are frequently membrane proteins, it would be most surprising if a high PUFA diet had no effect on the pharmacodynamic effects of drugs. For instance, an increase in membrane fluidity (induced by benzyl alcohol) has recently been shown to promote transcriptional upregulation of glucocorticoid receptor-dependent genes [47]. A few examples with PUFAs also exist in the literature. Thus, the essential omega-3 polyunsaturated fatty acid docosahexaenoic acid (DHA) exhibited a marked propensity to interact with both adenosine A2A and dopamine D2 receptors, leading to an increased rate of receptor oligomerization. It has been suggested that higher levels of PUFAs ameliorate neurodegenerative diseases which could potentially have important implications for drug therapy of neuropsychiatric conditions like schizophrenia or Parkinson’s disease [48]. In animal models, substitution with specific PUFAs has already shown protective effects against neurodegeneration [49]. Pardini found that an enhanced nutritional uptake of PUFAs, particularly long chained omega-3-FAs, increased membrane fluidity, increased activity of selected drug activating enzymes, altered signaling pathways important for cancer progression and enhanced transport capabilities for selective anti-cancer agents leading to their accumulation in the tumors [50]. These changes in tumor fatty acid composition resulted in enhanced sensitivity to chemotherapy, especially in tumor lines that are resistant to chemotherapy, suggesting that long chained omega-3 PUFA supplementation may be beneficial in tumor chemotherapy. More extensive studies of the interactions between PUFA supplementation and various drugs would seem to be warranted.

High protein meals also exert different effects on drugs. The blood flow in the GI tract increases upon a protein-rich meal and enhances drug absorption. However, protein-derived peptides might compete with drugs for transporter proteins leading to lowered absorption rates. This might be of relevance, for example, for the anti-Parkinson drug levodopa or beta-lactam antibiotics which are also dependent on transporter proteins for uptake [51]. On the other hand, proteins are suggested to increase intestinal transporters. Furthermore, protein-rich meals can have an impact on enzymes in drug metabolism and may alter the bioavailability of several drugs [41]. It has been shown that a switch to a high-protein/low-carbohydrate-containing diet can increase the clearance of beta-blocker propranolol by 74% and that of theophylline by 32% [52]. In contrast, high-carbohydrate/low-protein meals decrease the clearance of theophylline used in asthma therapy [53]. In clinical studies with patients with asthma or airway obstruction, a high-protein diet was associated with higher clearance rates of theophylline which affected the therapy [54,55]. Furthermore, changes in bioavailability have been described for the immunosuppressive agent tacrolimus which has a higher absorption rate after a high-protein meal as compared to a high-fat meal [56].

Carbohydrates are a heterogeneous group of food components which occur as mono-, di-, oligo- and polysaccharides, both digestible and non-digestible. Therefore, it is relatively difficult to clearly evaluate their effects on metabolism. As indicated before, a switch from low to high or high to low carbohydrate diet is associated with changes in drug clearance of theophylline by changes in CYP1A2 activity [53] and of phenytoin by alterations in CYP2C9 and CYP2C19 [57].

As alluded to previously, several studies show that the activity of the CYP system can be strongly altered by the composition of a meal in terms of protein, carbohydrate and fat content (summarized in [7]). The different macronutrients affected CYP450 enzymes differentially but the findings were not always conclusive. The activity of CYP1A2 seems to be increased by a high protein diet and fasting, but is reduced by high carbohydrate diet. In addition, the activity of CYP2C9 is decreased by fasting and increased by overnutrition. In contrast, CYP2C19 was shown to be non-susceptible to fasting or high protein diets [58,59]. Other groups investigated effects of fasting on drug metabolizing enzymes and found that a 36 h withdrawal of food and drink, except water did not significantly influence the activity of several CYP enzymes. However, CYP1A2, CYP2D6 and CYP 3A4 activities were significantly increased after fasting while CYP2C9 was decreased. These changes in drug metabolism were associated with increased caffeine, metoprolol and midazolam and decreased warfarin clearance and a modified efficacy of the drugs [58,60]. In addition to first phase metabolic enzymes, regulation of second phase enzymes (Uridine 5′-Diphospho-Glucuronosyltransferase (UGT)1A4/2B4/2B) was also observed after fasting, leading to decreased midazolam metabolism [60].

#### 3.2.2. Dietary Supplementation with Micronutrients and Bioactive Compounds

Micronutrients are essential dietary factors which are needed by human organisms for proper physiological functions. In contrast to macronutrients, micronutrients are required only in low amounts and comprise, for example, vitamins and minerals. They act mainly as enzymatic cofactors but are not needed for energy production and anabolism. Bioactive compounds are a class of non-essential micronutrients and comprise, among others, different polyphenols derived from plants (phytochemicals) [61,62].

There is an ongoing trend to supplement “normal” alimentation with vitamins or multivalent cationic minerals which might affect drug properties [63]. Minerals are reported to exhibit so-called antacid interaction leading to complex formation, changes in adsorption, gastric and urinary pH and clearance of drugs [64,65,66]. Similar to fruit juices, food supplements containing flavonoid or antioxidant components can induce changes in drug PK and PD. As an example, paracetamol, a potent antinociceptive and antipyretic drug, and statins, lipid-lowering drugs, can be modulated by antioxidative food supplements or phenolic phytochemicals [67]. A recent review of clinical interactions with CYPs identified CYP-based interactions between 261 herbal, food and dietary supplements and 117 anticancer drugs [68].

#### 3.2.3. Phytochemicals

Different polyphenols are produced by plants for protection against several environmental threats such as UV. The group of plant polyphenols comprises flavonoids, phenolic acids, coumarins, stilbenes and lignans. They are ingredients of vegetables, fruits and nuts and associated with beneficial effects on health [69]. These polyphenols are thus, taken up in the diet, for instance, as epicatechin in cocoa, epigallocatechin gallate in green tea, anthocyanins in berries or quercetin in onions. Plant polyphenols, in particular epicatechins, have also been associated with decreases in insulin resistance, possibly by interaction with beta-cell damaging reactive oxygen species, glucose transporters or through anti-inflammatory mechanisms [70]. These beneficial properties might increase the efficacy of therapeutic measures against the development of diabetes.

Flavonoids in food have positive properties in that they exert anti-inflammatory actions and hepatoprotective effects but they are also able to modulate metabolism, efficacy and toxicity of drugs, among other mechanisms by modulation of CYP450, glucuronyltransferases and transporter proteins [71]. Very common examples are bioflavonoids in fruit juices, particularly grapefruit juice, which alter CYP450 enzymes as well as several transporter proteins such as Pgp, multi–drug resistance proteins (MDR) or OATPs [72,73,74]. Several studies indicated that bioflavonoids such as naringin or naringenin in grapefruit juice lead to inhibition of CYP3A4 and OATP1 and 2. By inhibition of CYP3A4, they induce an increase in the bioavailability of drugs such as calcium channel blockers, benzodiazepines, and lipid-lowering statins which are CYP3A4 substrates. These modulations in bioavailability might then be associated with serious adverse effects, including changes in ECG, or rhabdomyolysis, a pathological breakdown of skeletal muscle [75,76]. Inhibition of OATPs by grapefruit juice is associated with decreased intestinal absorption of drugs acting as transporter substrates, such as antihypertensive ACE inhibitors [77]. Further studies showed that a number of other fruit juices (e.g., apple and orange) are also able to change the pharmacological effects of drugs by altering activity and expression of intestinal OATP1A2, CYP3A4 or Pgp or by direct interaction with the drugs [74]. However, other reports indicate that it is difficult to estimate the pharmacodynamic outcome of these effects due to a high variability of fruit juice composition and the differences among individuals [78,79]. Furthermore, the daily uptake of grapefruit juice is moderate in most countries and therefore, only a few cases might contribute to the clinical relevance of fruit juice–drug interactions [80].

A further important contribution to diet regimens to date can be attributed to soya products with a high isoflavone contents. Vegetarian and vegan diets as well as Asian foods are mainly based on soya and the number of people who replace meat by plant food is constantly increasing. Isoflavones may lead to modification of CYP enzymes and other cellular proteins involved in drug metabolism [81]. Clinical studies have shown that genistein, one of the main isoflavones in soya, induces a decrease in CYP1A2 and CYP2A6 activity [82,83] while causing a slight induction of CYP3A4 [84]. Flavonoids, at least in vitro, are able to counteract the generation in the liver of the reactive metabolite of paracetamol by inhibiting human hepatic CYPs, thereby reducing paracetamol liver toxicity [85].

An essential regulator of cellular antioxidant response is the transcription factor Nrf2. It is known as the master regulator of redox homeostasis and regulates nearly 600 genes involved in cellular protection against factors contributing to oxidative stress, including aging, disease and inflammation [86,87]. Flavonoids are not only antioxidant as a consequence of their ability to scavenge oxygen radicals, but more specifically through activation of Nrf2. This probably occurs by Michael addition between the polyphenols and cell surface thiol groups [86,88,89,90]. This activation of Nrf2 contributes to the anti-inflammatory activity of flavonoids and will modulate hepatic oxidative metabolism and potential hepatic toxicity of a number of different drugs [91]. Based on the same mechanism, it has been proposed, though, that flavonoids may also interfere with the effects of cancer chemotherapy [92]. It is often reported that vegetables from cruciferous plants (e.g., broccoli, spinach) contain indoles which can modify the metabolism of specific drugs. It is proposed that they modify CYP450 as well as phase II enzymes which might influence, for instance, the metabolism of phenacetin and paracetamol [93,94]. Furthermore, it was shown that diets containing broccoli or cabbage and Brussel sprouts increased elimination of caffeine metabolites (CYP1A2) [95]. Watercress decreased the activity of CYP2E1 and increased the bioavailability of the muscle relaxant chlorzoxazone, while decreasing the production of analgesic paracetamol metabolites [96,97].

#### 3.2.4. Minerals

Minerals are essential chemical elements which are required for the proper functioning of a living organism. They are taken up with food and comprise calcium (e.g., in dairy products), phosphorus (e.g., in meat), sodium (e.g., in table salt), potassium (e.g., in vegetables) and magnesium (e.g., in nuts) as well as trace elements such as iron, zinc or selenium. In addition to intake with food, these substances are frequently ingested by many people as food supplements. Several minerals are able to interact with drugs with different characteristics. Most interactions are known for calcium in milky products. Calcium can change the uptake of several drugs, such as the antibiotic fluoroquinolones or the antirheumatic/anticancer agent, methotrexate. Furthermore, milk inhibits the uptake of trace elements like iron. In addition, it is known that Ca^2+^, Mg^2+^ and Fe^2+^ can form complexes with drugs and thereby, change their solubility which has been shown for several antibiotics [98]. For this reason, the antibiotic tetracycline should not be given with dairy products, calcium or multivitamin supplements [99].

#### 3.2.5. Vitamins

The inhibitory effect of vitamin K-rich food (e.g., lettuce, avocado or liver) on the activity of warfarin, a common inhibitor of blood coagulation, is one of the best known examples of vitamin–drug interactions. Vitamin K is an essential component of the blood-clotting process which is inhibited by warfarin. Continuous uptake of vitamin K-rich foods can lead to warfarin resistance and to a requirement for dose adjustment [100,101]. Folic acids can also alter the metabolism of drugs. Thus, it has been shown that folic acid leads to a decrease in the plasma concentration of the anticonvulsant diphenylhydantoin [102,103]. Furthermore, vitamin C competes with estradiol for sulfate conjugation in the intestine and thereby, induces an increase in the bioavailability of estradiol which might impact on contraceptive drug efficacy [104,105]. In addition to these changes, vitamin C is suggested to be involved in a number of metabolic pathways, including glutathione recycling or catecholamine synthesis. By affecting the synthesis of neurotransmitters, it might also alter the need for analgesic drugs [106]. A and D vitamins are also associated with changes in CYP450 activity. Deficiency of vitamin A mainly inhibits the activity of various CYP enzymes, while supplementation with vitamin A enhances their activity [107]. Vitamin D may induce CYP3A4 which might be associated with reduced bioavailability of CYP3A4 substrates, such as ketoconazole or midazolam [108]. A wide variety of antioxidant dietary supplements, including vitamins A, E and C are also able to activate Nrf2, and will thus modify oxidative metabolism of drugs [109].

### 3.3. The Role of the Microbiome

The human microbiome describes the occurrence and variety of microorganisms in the human body. It comprises mainly bacteria, but also viruses, fungi and protozoa and colonizes several tissues, mostly gut, skin and nose. The microbiome shows marked inter-individual variability and is highly adaptive. In a physiological, healthy state, the microbiome contributes to body homeostasis, while disturbances of the homeostatic balance have been associated with diseases such as inflammatory bowel disease, atherosclerosis or Alzheimer’s disease [110,111].

A complex bidirectional interaction between several drugs and the gut microbiome is well established. Antibiotic and non-antibiotic drugs are both able to influence the microbiome of patients. However, the microbiome can also impact the clinical efficacy of drugs by enzymatically altering the structure and metabolism of the drug and thereby, its bioavailability, a phenomenon that has been entitled pharmacomicrobiomics. Germ-free animal models indicate that the microbiome can influence the gene expression of metabolic proteins and alter the efficacy of several drugs [112,113,114], thereby also contributing to pharmacological plasticity.

As mentioned above, most drugs are administered orally and have to pass the gastrointestinal tract where they come in contact with commensal microbes in the gut. Since microbes express a high variety of genes, the effects of drugs might be altered by the occurrence of drug-metabolizing enzymes produced by microbes or by direct interactions between microbes and drugs. These interactions may occur as direct effects of the microbial enzymes on drug metabolism or as indirect effects of microbial metabolites acting on signal transduction pathways of the host [115,116]. A study which investigated the metabolism of 271 drugs by human gut bacteria, revealed that about two-thirds of the drugs are metabolized by the bacterial strains. Microbiome-encoded metabolic enzymes contribute to the formation of the metabolites [117]. However, metabolic pathways catalyzed by hepatic enzymes of the host and enzymes of the microbiome are clearly different. In the liver, drugs are metabolized by oxidative and conjugative reactions while the gut microbiome induces hydrolytic and reductive reactions that contribute to drug metabolism [118]. It is also known that the microbiome can lead to individual-specific alterations in drug responses, as shown for instance, in cancer treatment with PD-1 immunotherapy [119,120].

Changes in the microbiome can be initiated by several factors including drug administration, circadian rhythm, exercise and diet. It is suggested that diet-induced modulation of the microbiome might contribute to beneficial effects of a diet in diverse diseases. So far, carbohydrates are the best studied nutrients, influencing the microbiome by serving as a major energy source for bacteria while functions of proteins and fat have gained less attention [121]. Dietary alterations can rapidly change the bacterial composition of the gut by promoting the growth of specific bacterial groups. For instance, diets containing plant proteins, unsaturated fats or probiotics, increased the quantity of the bacterial species *Lactobacillus* and *Bifidobacteria* which might contribute to beneficial health effects in a Mediterranean diet, while Western diet decreases these bacterial strains associated with the development of several diseases [111]. Some reports have already discussed whether these effects could be used as therapeutic measures for precision therapeutic nutrition. In addition, diet-induced changes in the microbiome have an impact on the immune system, an effect which might also be useful for personalized nutritional approaches [122,123]. However, it is not clear so far whether long-term dietary changes are able to induce a permanent shift in the microbial composition although there are data showing a change in gut microbial enterotypes after a long-term diet [124]. Several reports indicate that uptake of proteins is associated with a high diversity of microorganisms. However, this might lead, for instance, to an increased risk of developing inflammatory bowel disease, in particular with high consumption of animal proteins [125,126].

High uptake of saturated fatty acids is associated with a lower diversity of microbiota in humans, while PUFAs may increase the occurrence of several bacteria associated with anti-inflammatory and anticancer effects [127,128]. The amount of fat in the diet also shifts the composition of different microorganisms in the gut, which is associated with reduced concentrations of fasting glucose and cholesterol on a low-fat diet [129]. Carbohydrates also alter the microbiome in a variable manner depending on the carbohydrate type and the amount of uptake. Digestible carbohydrates are metabolized to glucose and stimulate the release of insulin while non-digestible carbohydrates such as fibers produce prebiotics. Prebiotics are associated with several health benefits induced by mechanisms involving the gut microbiome, such as improvements in metabolic disorders [130]. So far, there are only a few studies which have directly investigated food-induced changes in the microbiome and their impact on drugs. Current data available indicate that polyphenol-rich nutrition, for instance, influences a specific bacterial strain (*Akkermansia*) in the gut, thus potentially contributing to an increased response to antitumor therapy with PD-1 blocking antibodies [119,131].

### 3.4. The Role of the Immune System

The uptake of different types of nutrients not only affects the pharmacokinetics of drugs but may also indirectly alter drug responses by modulation of the immune system, for instance, by modifying the microbiome as mentioned above, potentially also affecting actions of immunomodulatory drugs. Many food constituents, including flavonoids, exert immunomodulatory and anti-inflammatory activities which contribute to the beneficial health effects of a balanced diet and of food supplements [132]. Zinc and selenium are trace elements in food which, among many other effects, are essential for healthy innate immune cell function by regulating T-cell signaling, phagocytes and NK cell activities and ensuring effective defense against pathogens and tumors [133,134]. In addition, zinc is a cofactor for thymulin which ensures normal differentiation and maturation of T lymphocytes. Selenium is essential for the activity of the hydroperoxide degrading enzyme, glutathione peroxidase which protects against oxidative injury to cells, including immune cells, and tissues. Vitamin E, which is active against oxidative damage to lipids and vitamin C which is a soluble antioxidant factor, also contribute to the protection of the organism against oxidative injury. Since vitamin C is a cofactor for the biosynthesis of CYPs, deficiency is clearly likely to have effects on the metabolism of a number of drugs. Drug interactions due to excessive vitamin C supplementation, on the other hand, are unlikely as it is rapidly excreted in the urine. Deficiencies in Vitamin E are associated with disturbances in innate and adaptive immunity [133]. D vitamins also affect both the innate and adaptive immune system, showing stimulatory effects on innate immune cells while T and B-cells are inhibited, which might reduce autoimmune reactions reinforcing responses to anti-inflammatory drug therapy.

Dietary fatty acids contribute to the cellular uptake of PUFA substrates for inflammatory lipids such as prostaglandins, leukotrienes and hydroxy-fatty acids and thereby to inflammatory responses [135]. On the other hand, some PUFAs, such as the omega-3-fatty acids in fish oil, lead to the formation of different types of prostaglandins and leukotrienes which are not inflammatory and promote inflammation resolution [13,136]. These diet-induced effects may then support—as with fish oil—or inhibit—as with other fatty acid oxidation products—drug efficacy. In addition, dietary PUFAs are incorporated into all cell membranes and enhance the fluidity of the membranes. As a consequence, responses of both the innate and adaptive immune systems are modified, epithelial barrier protection enhanced and the activation of nuclear and transmembrane receptors altered [137]. Such effects have been observed on cholesterol efflux from human macrophages and atherosclerosis [138], in relation to neurotransmitter signaling in both depression and schizophrenia [139,140], as well as on viral infection of cells [141], with inevitable modifying consequences for the efficacy of drug therapy.

In summary, it can be assumed that food habits vary tremendously between individuals, just taking into account the fact that different people eat meat, are vegetarians or vegans. Some people eat low-carb, others follow high protein diets, etc. Therefore, it is very difficult to provide an overview of all potential effects of diet on diverse drugs. A problem with food–drug interactions is that they are not generally predictable. There is a huge variability in drug formulations, characteristics of the meals and the time of drug intake in relation to the meal. In addition, the “reactive” ingredients in food are often not clear. So far, effects of food on drugs have mostly been evaluated in clinical studies which are cost intensive and take a lot of time. Furthermore, clinical studies often do not provide mechanistic insights into the food–drug interaction. Novel approaches suggest that it might also be possible to apply physiologically based pharmacokinetic (PBPK) models that combine drug properties and physiological data to predict mechanisms and potential clinical relevance of food effects. With these models, it should be possible to estimate the food effects and their magnitude with regard to pharmacokinetic parameters [142,143,144]. Although establishing these models affords in vitro and in vivo verification at the start, at later stages these approaches would provide large health benefits.

## 4. Exercise

Similarly to diet, exercise is often a basal therapy for several disturbances and can improve symptoms of cardiovascular diseases such as hypertension or high blood glucose levels in diabetes patients [145,146]. Furthermore, physical activity can ameliorate pathologies of the nervous and the immune system (such as Alzheimer’s disease or depression, inflammatory diseases) [10,11]. It is recommended by physicians, often in combination with a pharmacological therapy for the underlying disease(s). In common with diet, exercise and drugs also interact with each other in a bidirectional manner. Several medications may alter exercise performance, while exercise has an impact on the efficacy of drugs as well. However, the impact of exercise on the pharmacokinetics of drugs is complex and depends on the drug characteristics as well as on exercise-related conditions, such as mode, duration and intensity of exercise. Nevertheless, the impact of certain types of physical activity should be considered in relation to pharmacological therapy and in the development of novel drugs.

Physical activity (PA) can be roughly distinguished into acute and long-term (chronic) as well as aerobic or resistance exercise. These different forms of activities are associated with acute and chronic changes in physiology which can range from short time adaptation within minutes to hours, as well as long term adaptation over a period of days or weeks. The changes apply to cardiac output, body temperature, gastric motility, plasma volume, plasma proteins, body composition, heart and skeletal muscle capillarization, as well as tissue mitochondrial density. All these adaptations can affect drug pharmacokinetics and -dynamics. Although the awareness of these exercise properties is increasing, there are only limited data on the effects of exercise on drug efficacy in patients. Long term health benefits of exercise are often ascribed to changes in the expression and activity of proteins but these changes are often short-lived and maintained only for several hours after exercise. However, repeated exercise can also lead to accumulation of changes if the intervals between PA are short and the half-life of the effect is long [147]. In this regard, such exercise can have strong effects on the expression and activity of metabolic enzymes.

PA affects the pharmacokinetics of drugs in the exercising individual in terms of absorption, distribution, metabolism and excretion, and may thereby modulate C_max_, T_max_, t_1/2_ and F. One main factor determining exercise-induced adaptations is a change in blood flow distribution which affects the physiology of several organs and the blood composition. This may also have an impact on plasma protein binding of the drugs and may affect their efficacy, since only unbound drugs can induce an effect.

### 4.1. Exercise and Drug Absorption

During exercise, the cardiac output increases and blood flow is shifted to increased perfusion of skin and muscles, thus leading to decreased blood support of inner organs [148,149]. Depending on exercise intensity, this can lead to decreased gastric motility and emptying associated with delayed drug absorption. Furthermore, the increased transit time in the GI tract might support interaction of the drug with gastric acid secretion and enzymes which may lead to changes in solubility, metabolism and transporter influx/efflux. However, this is not the case in chronic aerobic exercise which induces a shortening of the transition of solid food through the stomach and an increase in GI permeability [150,151]. In any case, these changes can influence the bioavailability of drugs. Gastric acid secretion and concentrations of HCl are lower during exercise, leading to facilitated translocation of unionized basic drugs into the vasculature, while acidic drugs show higher rates of ionization and will thereby be retained in the GI to a higher degree. In addition to changes in the gastrointestinal tract, which will most probably affect absorption of orally administered drugs, the increased blood flow in the working muscles during exercise can have an impact on the absorption of subcutaneously or transdermally administered drugs, associated with increased skin temperature and increased hydration. For instance, it has been shown that subcutaneously injected insulin shows a higher absorption rate in working muscles resulting in further lowering of plasma glucose levels [152]. Moreover, transdermal absorption of nitroglycerin and nicotine from patches can be increased by exercise [153]. Intramuscular injections of penicillin and diazepam also delivered higher plasma concentrations after exercise in comparison to resting controls [154,155].

### 4.2. Effects on Drug Distribution

Several studies have also indicated drug distribution changes as a result of exercise. During acute exercise, the concentration of plasma proteins shows a relative increase due to a decrease in plasma volume [156]. On the other hand, regular aerobic exercise leads to an increase in the plasma volume [157]. Both conditions affect drug concentrations in the plasma as well as distribution and elimination by changes in plasma protein binding and distribution of the unbound drug [156]. Distribution might also be affected by the changes in blood flow which hinder the drug from reaching its target tissue. Several clinical studies indicate that beta-blocking drugs, for instance, show a change in their distribution during exercise, but it has not been clarified whether the changes are associated with modified drug action [158,159]. In addition to changes during acute bouts of exercise, chronic regular exercise leads to a switch from adipose tissue to fat-free body mass. Adipose tissue is less hydrated and perfused indicating that fat reduction increases bioavailability and distribution of drugs which are dependent on the physiochemical properties of the respective drug [13,18]. In addition, it is known that exercise induces release of free fatty acids (FFAs) from adipose tissue and their shift to the blood circulation. The FFAs can then competitively hinder drug–protein binding which has been shown in rats, for example, where exercise-induced FFA increases could be correlated with increased ampicillin levels in serum and tissues [160]. Clinical studies supporting these effects are still lacking. The distribution and metabolism of drugs is also affected by an exercise-induced increase in heart and skeletal muscle capillarity and in mitochondria [147]. The increased blood circulation enhances drug distribution while more mitochondria can affect the activity of drug metabolizing enzymes [13]. Both effects might occur concomitantly, since changes in blood flow are able to directly affect mitochondrial biogenesis and activity, as shown in studies using blood-flow restriction exercise [161].

### 4.3. Exercise and Drug Metabolism

As mentioned above, drug metabolism occurs mainly in the liver by the action of CYP450 enzymes. Changes in hepatic blood flow during exercise may influence hepatic metabolism and clearance of drugs. However, this is at least partially dependent on the drug hepatic extraction rate. Drugs with low hepatic extraction are only dependent on changes in blood flow to a limited extent, while metabolism of drugs with high hepatic extraction can be drastically modified by blood flow alterations [162]. Another factor is that acute exhaustive exercise is associated with several inflammatory effects [163,164] which may influence the metabolism of drugs, such as by downregulation of mRNA synthesis of metabolizing enzymes [14]. This might occur in association with epigenetic effects of exercise, as has been shown for adenosine monophosphate kinase, for example, a key metabolic enzyme, which showed modified methylation in blood and skeletal muscle cells after exercise [165]. In contrast, specific types of chronic exercise have been linked to upregulation of metabolizing enzymes, leading to increased clearance, potentially due to an increase in liver size and CYP450 content [166]. A further effect of exhaustive exercise is accumulation of lactate which decreases the blood pH. This might affect the metabolism of basic drugs which have an increased unbound fraction in acidic environment, while acidic drugs show a higher protein binding capacity (e.g., to albumin) [167].

### 4.4. Effects of Physical Exercise on Drug Excretion

Exercise can also affect excretion of drugs from the circulation which is mainly performed by urine and bile. Due to decreased blood flow in inner organs, the glomerular filtration rate in the kidney can decrease up to 30% during exercise performance depending on exercise intensity [156]. Therefore, drugs which are secreted in an unchanged form are most likely affected by exercise and may show an accumulation in the plasma as has already been shown for several drugs including atenolol or procainamide [168,169]. However, it has not been clarified whether the changes have clinical relevance. Exercise affects renal excretion, for instance, by a shift to a higher urinary pH. As mentioned before, this leads to neutralization of weakly basic drugs, limiting their excretion, while acidic drugs are ionized and are thus, more easily excreted [170]. Biliary excretion also changes with exercise. Bile acid flow and excretion is increased and intestinal reabsorption decreased, leading to higher excretion rates of cholesterol, for example [171].

### 4.5. Clinical Studies on Exercise and Drug Effects

Several clinical studies have been performed to investigate the effects of physical activity on drugs. Many of these studies focused on cardiovascular drugs, such as beta-blocking agents like atenolol or propranolol, the calcium channel blocker verapamil or digoxin. The results are relatively heterogeneous showing increased, decreased or unchanged drug plasma levels after exercise which might be due to the fact that study conditions differed drastically among each other. Healthy volunteers as well as patients on drug therapy were investigated and the modes of exercise comprised treadmill walking, cycling, stair climbing etc. over differing periods of time, with different intensity and various numbers of repetitions. Interestingly, in most cases, only pharmacokinetic parameters, such as plasma concentration or urinary excretion were determined, while clinical parameters such as blood pressure, in the case of beta-blockers, were not analyzed. Control groups were often not used for comparison and many of the studies included only low numbers of participants (reviewed in [6]). Further studies assessed the influence of exercise on the efficacy of warfarin. Similar to vitamin K rich food, the level of physical activity has an impact on warfarin effects in anticoagulation therapy. Highly active patients required higher doses of warfarin but showed a 38% lower risk of major hemorrhage. The mechanisms leading to these effects have not yet been clarified [172,173]. As mentioned previously, exercise also shows a strong effect on insulin-regulated blood glucose levels. Injection of insulin into different sites of the body was associated with an increase in insulin absorption, hyperinsulinemia and hypoglycemia in combination with physical activity [174,175,176]. Theophylline, which is used for the treatment of asthma patients, is also affected by exercise performance with increases in half-life and decreases in clearance, which might lead to toxic effects [177].

In spite of all the studies mentioned above, it is difficult to create a general model for exercise–drug interactions due to the fact that drugs as well as exercising individuals show heterogeneous properties with regard to their biochemical characteristics, for example, or the exercise type which is performed and the training status. This is even more complicated considering the administration routes of the drugs. Nevertheless, these studies at least indicate that exercise can affect the pharmacokinetics of drugs (e.g., some beta-blockers) with a narrow therapeutic index (ratio between beneficial and toxic doses), which is an important consideration when treating exercising patients with these drugs. In addition, diabetic patients on insulin need to be treated with caution and should be advised to monitor their blood glucose frequently during exercise.

In summary, it is well established that exercise can influence the pharmacokinetics of drugs [178], but the potential impact on therapeutic responses is still less clear. Currently, standardized clinical studies on these therapeutic changes are largely lacking, although an understanding of these drug–exercise interactions is extremely important, especially for patients with existing conditions on drug therapy with frequently prescribed or narrow therapeutic range drugs. Furthermore, such insight could support the efficacy and also the safety of newly developed drugs. The problem with exercise, as with diet, is its high heterogeneity with regard to the level of exhaustion, and whether aerobic or anaerobic exercise, endurance or strength training is exerted. Furthermore, it is not known which and how many molecular responses are induced by exercise and which are regulated by other biological conditions, such as age, gender, genetic precondition and nutrition, thus making a distinction of sport effects on drug efficacy even more complicated. Nonetheless, the pharmacokinetic parameters of most medications are typically assessed under resting and non-stressful conditions, and consequently the potential for drug–exercise interactions may have been underreported. Clinical studies integrating physical activity are mostly performed in young healthy volunteers who exert single bouts of a specific type of exercise to achieve some standardization of the studies. However, these study conditions do not mirror a realistic clinical situation and therefore, the clinical significance is often questionable. It would be extremely important to also perform these studies in patients with different diseases and on different drug therapies, but under standardized conditions for the exercise program. Furthermore, studies on different types of long-term continuous exercise are needed, since first hints indicate that the duration of exercise may make a large difference [166,179].

Standardization of exercise conditions in clinical studies might be facilitated by clearly defined study protocols and population subsets. Furthermore, a combination of studies performed at different research institutions would clearly advance our knowledge on exercise effects. Then it might also be possible to adjust therapies individually for different patient populations by distinguishing between physically active and inactive individuals, for instance, and integrating their fitness level as well as the intensity and type of exercise.

## 5. Concluding Remarks

Exercise and dietary changes are often recommended as basic or concomitant therapeutic measures in several diseases. Their beneficial properties are undoubted but their potential effects on drug efficacy are often not respected, although they may have an impact on the therapeutic outcome in individual patients. Several scientific approaches have tried to clarify pharmacodynamic and pharmacokinetic changes induced by exercise and dietary interventions, but there are still many open questions left. A big problem in this context is the high variability of lifestyle-induced effects. It is obvious that exercise and diet are parameters which are highly variable and depend on a large number of different prerequisites. Interactions between food, exercise and drugs are almost limitless and depend on type of food/exercise, strength, route of administration of the drug, the drug chemical properties, genetic predisposition or microbiome of patients, to give some examples. Exercise- or diet-induced physiological changes can be short- or long-term and might affect the food–drug or exercise–drug interactions, probably to some degree through alterations in the gut microbiome which has rarely been investigated so far. A further point is that effects differ largely from drug to drug and effects of single drugs or drug classes do not necessarily reflect effects on other drugs. Since it might not be possible to comprehensively clarify all diet–drug, exercise–drug interactions, the occurrence of pharmacological plasticity might be overcome by applying several different alternative measures, such as increasing the specificity of newly developed drugs for patient sub-populations. A further possible solution to optimize the therapy under lifestyle changes would be a multifactorial approach using sequential therapeutic approaches with different drug types to determine which treatment provides the optimal therapeutic outcome [1]. In any case, it must be taken into account that drug dosage may need to be adjusted after or during lifestyle changes, as different types of pharmacological plasticity lead to changes in drug efficacy and toxicity. This is particularly important for drugs with a narrow therapeutic window. Patients taking such drugs should be informed and carefully monitored. On the other hand, it should also be kept in mind that, in some cases, lifestyle-induced effects on drug responses may be statistically significant but have limited clinical relevance.

In conclusion, data summarized in this review indicate that lifestyle changes in diet or physical activity induce a wide spectrum of pharmacological plasticity which might interfere with the efficacy and toxicity of several drugs. These potential interactions should be considered by physicians and patients as well as during the development of novel therapeutic drugs.

## Figures and Tables

**Table 1:** Physiological changes induced by food and their effects on pharmacokinetics.

Changes in Physiology	Potential Effects	PK Effect	Examples	Ref.
Reduced gastric emptying	Decreased transport of drug to intestine	Increased T_max_	NSAIDs	[20,21]
Increased blood flow in GI tract	Saturation of liver enzymes, avoidance of first pass metabolism	AUC and Cmax increase	Propranolol	[22]
Increased pH in stomach	Altered solubility of drugs	AUC and C_max_ for acids increase AUC and C_max_ decrease for basic drugs	Cefuroxime, Dipyramidol	[20]
Food ingredients alter solubility of drugs (e.g., lipids)	Lipophilic drugs show increased solubility	AUC and C_max_ increase	Fenofibrate	[23]
Inhibition of GI enzyme or transporter activity	Decrease in drug metabolism, decreased efflux Decreased drug uptake	AUC and C_max_ increase AUC and C_max_ decrease	Sirolimus, Midazolam Fexofenadine	[24,25]

## Data Availability

PubMed Database, Citation lists of initially read publications.

## Abbreviations

ADME	Adsorption, Distribution, Metabolism, Elimination
Cyp	Cytochrome
FDA	Food and Drug administration
FFA	Free fatty acid
GI	Gastrointestinal
GPCR	G-Protein coupled receptor
PD	Pharmacodynamics
PK	Pharmacokinetics
Pgp	P-glycoprotein
PUFA	Polyunsaturated fatty acids
